# Cost evaluation of PAGE-B risk score guided HCC surveillance in patients with treated chronic hepatitis B

**DOI:** 10.1186/s12913-021-06794-6

**Published:** 2021-08-21

**Authors:** Martin F. Sprinzl, Christina Feist, Sandra Koch, Wolfgang M. Kremer, Karl J. Lackner, Arndt Weinmann, Peter R. Galle

**Affiliations:** 1 Medical Department I, University Medical Center of the Johanne Gutenberg University Mainz, Langenbeckstrasse 1, 55131 Mainz, Germany; 2 Institute for Laboratory Medicine and Clinical Chemistry, University Medical Center of the Johanne Gutenberg University Mainz, Langenbeckstrasse 1, 55131 Mainz, Germany

**Keywords:** Hepatitis B, Hepatocellular carcinoma, PAGE-B, Score, Costs

## Abstract

**Background:**

The PAGE-B score (Platelet Age GEnder–HBV) selects chronic hepatitis B (cHB) patients showing no relevant 5-year risk for hepatocellular carcinoma (HCC). We, therefore, explored potential cost reduction following the introduction of a PAGE-B tailored ultrasound screening in a single center cohort of cHB patients receiving stable antiviral therapy.

**Methods:**

cHB patients attending throughout the year 2018 were documented. Patients eligible for PAGE-B score were classified into high (≥18 points), intermediate (10–17 points) and low (≤9 points) HCC risk groups. Patients of the low HCC risk group could postpone HCC screening to reduce HCC screening expenses. Full costs for hepatic ultrasound were assessed.

**Results:**

Throughout the year cHB patients (*n* = 607) attended our clinic, which included PAGE-B eligible patients (*n* = 227, 37.4%) of whom *n* = 94 (15.8%) were allocated to the low HCC risk group. Sonographic HCC screening during a median exam time of 12.4 min (IQR 9.2–17.2) resulted in total costs of 22.82 Euro/exam. Additional opportunistic expenses caused by patient’s lost earnings or productivity were 15.6–17.5 €/exam and 26.7 €/exam, respectively. Following a PAGE-B tailored HCC screening at our institution annual full costs for cHB patients could be reduced by 15.51%, which equals a cost reduction by 1.91% for our total sonography unit. In comparison, 1.35% up to 7.65% of HBV-infected patients of Caucasian descent could postpone HCC screening according to population-based estimates from Germany.

**Conclusions:**

PAGE-B risk score adapted screening for HCC is an efficient and cost neutral tool to reduce costs for sonography in Caucasian patients with chronic hepatitis B receiving antiviral treatment.

**Supplementary Information:**

The online version contains supplementary material available at 10.1186/s12913-021-06794-6.

## Background

Patients suffering from chronic Hepatitis B (cHB) develop a relevant morbidity and mortality caused by hepatocellular carcinoma (HCC) [1]. Surveillance by ultrasonography has therefore been established in cHB patients and improves overall survival of cHB patients [2]. Therefore, cHB treatment guidelines recommend HCC surveillance in all patients with liver cirrhosis every 3 to 6 months. In cHB patients without liver cirrhosis a diagnostic screening is generally recommended annually [3, 4].

Additional risk stratification, however, has identified cHB patients with a considerably lower cumulative HCC-incidence, who might not require HCC surveillance. Particularly antiviral therapy with nucleos(t)ide analogues (NA) has reduced the HCC risk of cHB [5, 6], which remains only minimally higher compared to hepatitis B virus carriers without disease activity [7]. This uncertainty of residual HCC development in NA treated patients, was addressed by the PAGE-B risk score (**P**latelet **A**ge **GE**nder–H**B**V) integrating age, gender and thrombocyte count, which selects primarily Caucasian patients with low HCC risk [8–10]. According to the PAGE-B score patients of the low HCC risk group (≤9 points) do not develop HCC under stable NA therapy during a 5-year follow up [8]. The large body of evidence has led to the recommendation of the European Association for the Study of the Liver (EASL), that cHB patients categorized into the low PAGE-B risk group could postpone HCC surveillance [11].

Given this new clinical data, it became possible to optimize allocation of clinical resources for HCC surveillance in cHB patients. However, the cost reduction for HCC surveillance, following a PAGE-B guided screening has not been defined. Hence, we explored the proportion of PAGE-B eligible cHB patients and the corresponding sonography costs to characterize the economic potential of PAGE-B adapted HCC screening.

## Methods

### Data acquisition and patient selection

For this observational single center study patient data were retrospectively retrieved from the hospital information system. Chronic cHB patients attending the liver disease out-patient clinic throughout the year 2018 were identified by a positive serum HBs-antigen (HBsAg) and cHB complications such as liver cirrhosis and hepatocellular carcinoma (HCC) were documented. Patients coinfected with hepatitis C, hepatitis D and human immunodeficiency virus were excluded. The PAGE-B score was calculated based on age, gender and thrombocyte count. PAGE-B strata were classified into high (≥18 points), intermediate (10–17 points) and low (≤9 points) HCC risk groups (additional Table 1) [8]. Patients eligible for PAGE-B score assessment had to receive effective antiviral therapy with second generation NA including entecavir (ETV), tenofovir disoproxil fumarate (TDF) and tenofovir alafenamide fumarate (TAF) for at least 1 year. The trial was conducted according to the principles of the Declaration of Helsinki. Approval was provided by the local ethic committee (State Chamber of Medicine, Rhineland-Palatinate, Germany, ethics approval number: 2019–14,206) and by the data safety officer (University Medical Center, Mainz, Germany).

### Diagnostic ultrasonography and time acquisition

HCC screening included an ultrasonography of the liver, spleen and adjacent lymph nodes. Each ultrasonography throughout April and May 2019 was monitored with an on-site time tracking device (Timeular® cube) by the examiner himself. The time span for each ultrasound (total exam time) was analyzed. In parallel, the total turnaround time spent at the ultrasound unit was documented for each patient at the out-patient clinic front desk. A patient flow chart of the HCC screening is provided (additional Fig. 1**)**.

A total of *n* = 268 sonographies were assessed. Eventually, *n* = 147 (54.9%) exams allowed detailed time assessments, which included selective liver ultrasound in *n* = 118 patients (44.0%). The exams were frequently performed by a specialist of internal medicine, who provided eleven years of work experience. A smaller proportion of exams (24.5%, *n* = 36/147) were provided by an assistant doctor with 3 years of work experience.

### Diagnostic ultrasonography expenses

The instrument expenses were 88,000 Euro (€) based on an updated sonography unit (Hitachi Arietta V70) meeting modern standards for HCC screening. The yearly costs were calculated by linear depreciation over a period of 5 years as defined by the German tax legislation (http://geman-taxes.de/pdf/AfA.pdf). Expenses for instrument services were derived from the service contract (3500 €/a). Facility expenses, including room rent, energy supply, water supply and cleaning services, were covered by the institutional allowance over 140.91 € per squaremeter per year. Consumable costs were based on listed prices as provided by the institutional purchase department. Work place software licenses for administration (i.s.h.med®, SAP), picture archiving and communication (ViewPoint®, GE healthcare IT) were included.

Average personnel costs were derived from the staff roster of the past 2 years. The team involved in HCC screening included one medical doctor performing the exam, one nurse providing medical assistance and two healthcare assistants providing administration. Labour costs were based on the collective bargaining agreement for public service employees (38.5 working hours/ week) amended at the 11.02.2015 (E&E-TV UM). Wage labour costs for medical staff (42 working hours/ week) were based on the collective bargaining agreement amended at the 01.01.2015 (TV-Ärzte/Universitätsmedizin).

### Biostatistics

Descriptive data are given in median and interquartile range (IQR) throughout the manuscript if not specified accordingly.

## Results

### Patient characteristics and PAGE-B score

A cohort of *n* = 607 patients with positive serum HBsAg were identified during the year 2018, who underwent laboratory work up and regular HCC screening during a total *n* = 1.210 visits per year. Patients with confirmed cHB receiving second-generation NA for a minimum of 1 year (*n* = 227) were eligible for HCC risk assessment by the PAGE-B score. This led to the identification of patients of high (*n* = 33), intermediate (*n* = 100) and low (*n* = 94) HCC risk, respectively (Table 1). Throughout the year, three patients of the total cHB cohort died, whereas no mortality was observed in the low HCC risk subpopulation. Mortality was caused by myocardial infarction (n = 1), terminal liver cirrhosis (n = 1) and hepatocellular carcinoma (n = 1). Patients with low HCC risk did also not develop any HCC during a median follow up of 21 months (IQR 19–25 months). The cumulative HCC incidence of the low, intermediate and high risk group was 0.0, 0.6 and 3.2% per year, respectively. The patient numbers with a low-risk PAGE-B score (≤9 points) were applied to calculate cost reduction by PAGE-B adapted HCC screening.

**Table 1:** Characteristics of chronic Hepatitis B patients

Population	Total (n = 607)	PAGE-B eligible (n = 227)	PAGE-B eligible & low risk (***n*** = 94)
Variables	Median (IQR), N (%)		
Age (years)	46.7 (37.2–57.0)	50.8 (41.8–59.4)	42.9 (33.6–54.0)
Age > 65 years	74 (12.2%)	34 (15.0%)	6 (6.4%)
Gender (male/female)	361/246 (59.5/40.5)	143/84 (63.0/37.0)	27/67 (28.7/71.3)
HBsAg positive	607 (100)	227 (100)	94 (100)
HBsAg (IU/l)	1665 (358–5222)	1714 (556–4909)	3700 (1199–8527)
Anti HBc-Ab	579 (95.5)	222 (97.8)	91 (96.8)
HBeAg positive	50 (8.3)	29 (12.8)	16 (17.0)
Anti HBe-Ab	517 (85.3)	179 (78.9)	76 (80.9)
HBV DNA < 20 U/l	331 (54.6)	197 (86.8)	74 (78.7)
**Ethnicity**			
Caucasian	519 (85.5%)	211 (92.9%)	86 (91.5%)
Asian	44 (7.2)	14 (6.2%)	7 (7.4%)
African	32 (5.3%)	2 (0.9%)	1 (1.1%)
not rated	12 (2.0%)	0 (0.0%)	0 (0.0%)
**Laboratory variables**			
ALT (U/l)	29 (21–40)	29 (23–34)	28 (20–28)
AST (U/l)	28 (24–35)	29 (21–41)	27 (22–33)
Total Bilirubin (mg/dl)	0.45 (0.62–0.85)	0.62 (0.45–0.92)	0.56 (0.40–0.78)
Albumin (g/l)	40 (38–42)	40 (38–42)	30 (38–42)
Quick (%)	96 (87–104)	96 (88–105)	96 (90–108)
Thrombocytes (× 10^9^/l)	219 (175–262)	229 (186–265)	247 (227–286)
**Clinical variables**			
Deceased	3 (0.5)	1 (0.4)	0 (0.0)
HCC	7 (1.2)	1 (0.4)	0 (0.0)
Liver stiffness (kPa)	4.8 (4.0–6.5)	4.9 (4.1–6.7)	4.5 (3.8–6.2)
Liver stiffness (> 10 kPa)	17 (2.8)	12 (5.3)	2 (2.1)
**Risk Scores**			
PAGE-B Score	10 (6–14)	10 (6–10)	6 (4–8)
≤ 9 Points	252 (41.6)	94 (41.4)	94 (100)
10–17 Points	273 (45.0)	100 (44.1)	–
≥ 18 Points	81 (13.4)	33 (14.5)	–
**Antiviral Therapy**			
Entecavir	110 (18.2)	105 (46.3)	41 (43.6)
TDF	118 (19.5)	118 (52.0)	49 (52.1)
TAF	4 (0.7)	4 (1.8)	4 (4.3)
Lamivudine	14 (2.3)	–	–
Adefovir	11 (1.8)	–	–
Telbivudine	1 (0.2)	–	–
Interferon-alpha	3 (0.5)	–	–
no antiviral treatment	345 (56.9)	–	–

Patient characteristics with confirmed chronic hepatitis B (n = 607). The subgroups of PAGE-B eligible patients (n = 227) as well as PAGE-B eligible patients with low HCC risk (n = 94) are shown. All patients attended the infectious and liver disease out-patient clinic during the year 2018. *IQR* Interquartile range, *TDF* Tenofovir disoproxil fumarate, *TAF* Tenofovir alafenamide fumarate

### Time requirements of sonographic HCC screening

The examiner and assistant staff were occupied for a median total exam time of 12.4 min (IQR 9.2–17.2 min) during liver ultrasound. This included a hands-on time of 5.4 min (IQR 4.1–7.8 min) for the examiner. The remaining time span was used for room preparations and documentation of the findings. The total exam time of the liver ultrasound was selected for subsequent work cost calculations during HCC screening. An administrative time of 30 s were estimated for the out-patient clinic as well as the endoscopy ward, respectively.

In parallel, patients were involved in HCC screening during a median total turnaround time of 45.0 min (IQR 34.0–59.8 min). The median total turnaround time was applied for calculation of external opportunistic costs for sonographic HCC screening.

### Full cost calculation of sonographic HCC screening

Full cost calculation for sonography included costs for instruments, software, technical services, facilities and consumables (Table 2). Average personnel costs expenses for administration (0.346 €/min), medical assistance (0.418 €/min) and physicians (0.574 €/min) were adjusted to the median total exam time and administration time as outlined above.

**Table 2:** Expenses for the sonography unit

**Facility expenses**^a^	**Euro/m**^**2**^**/year**	**Euro/room**^a^**/year**
Facility fee	67.8	10,712.4
Facility services	62.76	9916.08
Energy supply	4.98	786.84
Water supply	3.68	581.44
**Consumable expenses**	**Amount/exam**	**Euro/exam**
Paper cover (n)	1	0.08
Paper towels (n)	6	0.08
Desinfection towel (n)	1	0.05
Sonography gel (g)	4.9 g	0.01
Disposable gloves (n)	(2)	0.07
Print out (Paper/Toner) (n)	1	0.01
**Fixed costs**	**Amount**	**Cost (Euro)**
Computer^b^	1	625.94
Printer hardware	1	143.72
Administration workplace license	1/year	1904
Viewpoint workplace license	1/year	500

^a^Facility expenses were calculated on basis of the sonography room (15.8 m^2^). Consumable spendings and IT-support costs were derived from the institutional listed prices. ^b^Including operating system software license

Full costs calculation for HCC screening eventually resulted in a total of 22.82 €/exam (Table 3)**.** A capacity utilization of 75% was applied for the diagnostic sonography unit, as our institution runs two additional work places, used as back up for diagnostic or interventional sonographies. The capacity utilization grade was applied to correct for fixed costs, whereas consumable costs and personnel costs were purely based on exam numbers. A yearly interest rate was applied to account for general price increases as well as personnel expenses. The approximated yearly inflation rates were obtained from the German federal office for statistics survey [12].

**Table 3:** Full cost calculation for a single liver sonography (75% capacity utilization)

	Costs per exam					Factor
	2018	2019	2020	2021	2022	
**Consumables costs**						
Paper cover	0.080	0.081	0.082	0.082	0.083	1.01
Paper towels	0.080	0.081	0.082	0.082	0.083	1.01
Desinfection towel	0.050	0.051	0.051	0.052	0.052	1.01
Sonography gel	0.010	0.010	0.010	0.010	0.010	1.01
Disposable gloves	0.070	0.071	0.071	0.072	0.073	1.01
Print out (Paper/Toner)	0.010	0.010	0.010	0.010	0.010	1.01
**Fixed costs**						
Sonography instrument	3.755	3.755	3.755	3.755	3.755	1.00
Instrument Service	0.747	0.747	0.747	0.747	0.747	1.00
Computer hardware	0.134	0.000	0.000	0.000	0.000	1.00
Printer hardware	0.031	0.000	0.000	0.000	0.000	1.00
SAP workplace license	0.406	0.406	0.406	0.406	0.406	1.00
Viewpoint workplace license	0.107	0.107	0.107	0.107	0.107	1.00
Facility fee	2.285	2.308	2.331	2.355	2.378	1.01
Facility services	2.115	2.137	2.158	2.180	2.201	1.01
Energy supply	0.168	0.171	0.175	0.178	0.182	1.02
Water supply	0.124	0.125	0.127	0.128	0.129	1.01
**Personnel costs**						
Administration (out-patient clinic)	0.209	0.213	0.218	0.222	0.226	1.02
Administration (endoscopy ward)	0.137	0.140	0.142	0.145	0.148	1.02
Procedure (medical staff)	7.116	7.258	7.404	7.552	7.703	1.02
Procedure (assistent staff)	5.187	5.291	5.397	5.505	5.615	1.02
**Total costs**	**22.820**	**22.961**	**23.271**	**23.587**	**23.908**	

Full cost pricing of diagnostic liver sonographies was based on a total median exam time of 12.4 min as identified for liver sonography. The full costs were calculated on basis of *n* = 6250 sonographies per year at a capacity utilization of 75%. Factor, inflation rate

### Opportunistic expenses for sonographic HCC screening

Opportunistic costs result from patients lost income and lost productivity during HCC screening. The income calculation is based on the assumption, that cHB patients are typically fully integrated in the employment market. This particularly holds true for cHB patients without disease activity and no impairment of liver function, as observed in our cohort (Table 1).

German federal office income statistics were applied and adjusted to the median age of male patients (32.7 years, IQR 31.1–35.2 years) and female patients (49.9 years, IQR 38.9–56.4 years) from the cHB cohort [13]. According to available data (year 2014) an average gross income of 19.13 €/hour for men and 17.08 €/hour for women was extrapolated. The resulting income loss was 17.5 €/exam for male patients and 15.6 €/exam for female patients for the year of assessment. Finally, German unemployment rates of 4.1% for men and 3.3% for women as well as an annual wage increase of 2% as published by the German federal agency were taken into account [14]. Unemployed patients were not considered for calculation of lost income. Income of retired patients (age > 65 years) was assumed to be 15.5% of working persons income (age 15–64 years), due to paid activities at older ages [15] (Table 4).

**Table 4:** Opportunistic wage expenses by patient involvement

PAGE-B eligible cHB cohort (PAGE-B score ≤ 9 points)					
	2018	2019	2020	2021	2022
**Patients (n)**	**94**	**91**	**87**	**83**	**81**
**Male patients (n)**	27	27	25	25	23
Employed male patients (n)	26	26	24	24	22
Male patients, age < 65 years (n)	26	26	24	24	22
Male patients, age > 65 years (n)^a^	0	0	0	0	0
Average gross income / hour	19.1	19.5	19.9	20.3	20.7
Average gross income / minute	0.32	0.32	0.33	0.34	0.34
including ancillary labor costs/ minute	0.39	0.40	0.41	0.41	0.42
Opportunistic wage expenses / exam	17.5	17.9	18.3	18.6	19.0
Total costs (male patients)	454.0	463.5	437.2	446.5	419.0
**Female patients (n)**	67	65	62	59	58
Employed female patients (n)	65	63	60	57	56
Female patients, age < 65 years (n)	61	59	56	54	52
Average gross income / hour	17.1	17.4	17.8	18.1	18.5
Average gross income / minute	0.29	0.29	0.30	0.30	0.31
including ancillary labor costs/ minute	0.35	0.36	0.36	0.37	0.38
Opportunistic wage expenses / exam	15.7	16.0	16.3	16.7	17.0
Total costs (female patients, age < 65 years)	953.0	941.1	917.7	893.0	888.9
Female patients, age > 65 years (n)	4	4	4	3	3
Average gross income / hour	2.6	2.7	2.7	2.8	2.9
Average gross income / minute	0.04	0.04	0.05	0.05	0.05
including ancillary labor costs/ minute	0.05	0.05	0.06	0.06	0.06
Opportunistic wage expenses / exam	2.4	2.5	2.5	2.6	2.6
Total costs (female patients, age > 65 years)	9.4	9.3	9.0	8.8	8.8
Total costs (all female patients)	962.4	950.4	926.8	901.8	897.7
**Total costs**	**1416.4**	**1413.8**	**1363.9**	**1348.3**	**1316.7**

Opportunistic, age and gender adjusted costs of sonographic HCC screening during a median turnaround time of 45 min in PAGE-B eligible cHB patients with low HCC risk. All patients attended the infectious and liver disease out-patient clinic during the year 2018. Unemployment rates of 4.1% for men and 3.3% for women were considered as published by the German federal agency [14]. An annual income increase of 2% was applied. Unemployed patients were excluded from income calculations. Income of retired patients (age > 65 years) was assumed to be 15.5% of a working persons income (age 15–64 years) [15]. ^a^ Male patients age > 60 years are excluded by PAGE-B score ≤ 9 points

Gross domestic product (GDP) per working hour was also considered, as wages do not directly reflect overall productivity. Therefore, the average GDP of 35.56 €/hour, from the year 2017, was adjusted to the total turnaround time (45 min), resulting in a GDP loss of 26.7 €/ exam [16, 17]. Unemployed and retired patients were excluded from GDP calculation, as GDP reflects overall average work force productivity (age 15–64 years) of an economic region [16, 17].

### Cost reduction by PAGE-B score adapted HCC screening

The annual cost reduction at our institution was calculated on the basis of the full costs for liver sonography and the number of cHB patients with a low risk PAGE-B score (≤9 points) receiving NA treatment. This assessment led to a cost reduction of 2145 € for HCC screening during the year 2018 (Table 5). Given that only age is a time dependent variable of the PAGE-B score, whereas gender and thrombocyte count remaining unchanged, we extrapolated the number of annual HCC screens until a PAGE-B score of 10 points was reached. Based on these assumptions a median of *n* = 12 (IQR 6–12) postponed sonographies per person was calculated for our cohort, which makes a total of *n* = 1410 sonography screenings in total. A more restrictive calculation for a maximum 5-year follow-up, identified a total of *n* = 436 postponed HCC screenings. This resulted in a total full cost reduction of 10,488 € for a 5-year period (Table 5).

**Table 5:** Annual full cost reduction for PAGE-B tailored liver sonography

	Total costs reduction for cHB patients (PAGE-B score ≤ 9 points)					Factor
	2018	2019	2020	2021	2022	
**Patients (n)**	94	91	87	83	81	
**Consumables costs**						
Paper cover	7.52	7.37	7.13	6.81	6.72	1.01
Paper towels	7.52	7.37	7.13	6.81	6.72	1.01
Desinfection towel	4.70	4.64	4.44	4.32	4.21	1.01
Sonography gel	0.94	0.91	0.87	0.83	0.81	1.01
Disposable gloves	6.58	6.46	6.18	5.98	5.91	1.01
Print out (Paper/Toner)	0.94	0.91	0.87	0.83	0.81	1.01
**Fixed costs**						
Sonography instrument	352.94	352.94	352.94	352.94	352.94	1.00
Instrument Service	70.19	70.19	70.19	70.19	70.19	1.00
Computer hardware	12.55	0.00	0.00	0.00	0.00	1.00
Printer hardware	2.88	0.00	0.00	0.00	0.00	1.00
SAP workplace license	38.18	38.18	38.18	38.18	38.18	1.00
Viewpoint workplace license	10.03	10.03	10.03	10.03	10.03	1.00
Facility fee	214.82	216.97	219.14	221.33	223.54	1.01
Facility services	198.85	200.84	202.85	204.88	206.93	1.01
Energy supply	15.78	16.09	16.42	16.75	17.08	1.02
Water supply	11.66	11.78	11.89	12.01	12.13	1.01
**Personnel costs**						
Administration						
out-patient clinic	19.65	19.38	18.97	18.43	18.31	1.02
endoscopy ward	12.88	12.74	12.35	12.04	11.99	1.02
Diagnostic procedure						
medical staff	668.90	660.48	644.15	626.82	623.94	1.02
assistant staff	487.58	481.48	469.54	456.92	454.82	1.02
**Total costs**	**2145.08**	**2118.76**	**2093.26**	**2066.05**	**2065.26**	

Annual full cost pricing was based on a total median exam time of 12.4 min as identified for liver sonography a capacity utilization of 75%. The full costs were calculated on the basis of patients, who did not require sonography HCC screening according to a low PAGE-B risk score of ≤9 points. Factor, inflation rate

Nationwide cost reduction by PAGE-B adapted HCC screening was derived from population-based source data (additional Table 2), reporting cHB prevalence rates of 0.3% up to 0.7% in Germany [18–21]. The number of eligible patients was further adjusted by the rate (85–93%) of Caucasian HBsAg positive patients (Table 1) [22]. Treatment criteria according to management guidelines were considered, as only patients under second-generation NA therapy are eligible for PAGE-B scoring [3]. Due to the limited population-based data on cHB and liver fibrosis in Germany, only a relevant HBV viral load (> 2000 IU/ml) in 14.7 to 31.4% and an elevated ALT activity in 43.8 to 59.4% were considered among HBsAg-positive patients [19, 22, 23]. These data resulted in an estimated number of 13,169 up to 97,393 HBsAg-positive Caucasian patients with antiviral therapy indication in Germany [19, 22, 23]. Corresponding annual NA therapy costs for a total of 7,475,132 treatment days resulted in coherent patient numbers (*n* = 20,480) in Germany [24]. Eventually the proportion of a low risk PAGE-B score (≤9 points) was derived from two trials and from our cHB population, resulting in 24.7% [8], 38.6% [9] and 44.1% (Table 1), respectively. This range eventually results in a total number of 3253–42,950 NA treated cHB patients with low HCC risk in Germany.

The estimated population-based costs reduction for HCC screening in Germany was 154,116 – 2,034,951€ per year, based on the full costs for liver sonography and the loss of GDP (Table 6). Extrapolation for a 5-year period, which covers an average of 4.63 postponed sonographies per patient, resulted in a cost reduction (incl. GDP) of 731,557 € up to 9,421,823 € for the German population.

**Table 6:** Screening costs for hepatitis B patients (PAGE-B ≤ 9 pts.) in Germany

PAGE-B eligible cHB cohort (PAGE-B score ≤ 9 points)					
	N_**min**_	N_**max**_	Costs/Exam	Cost_**min**_	Cost_**max**_
**Sonography full costs (Euro)**	3253	42,950	22,8	74,164	979,262
**Opportunistic wage costs (Euro)**	N_**min**_	N_**max**_	Costs/Exam	Cost_**min**_	Cost_**max**_
**Male patients (n)**	934	12,327	–	–	–
Employed male patients (n)	895	11,821	–	–	–
Employed male patients, age < 65 years (n)	895	11,821	17,50	15,667	206,872
**Female patients (n)**	2319	30,623	–	–	–
Employed female patients (n)	2243	29,613	–	–	–
Employed female patients, age < 65 years (n)	2099	27,718	15,70	32,957	435,167
Employed female patients, age > 65 years (n)	134	1774	2,40	322	4257
Total wage costs	–	–	–	48,947	646,297
**Sonography costs incl. Opportunistic wage loss**	**–**	**–**	**–**	**123,111**	**1,625,559**
**Opportunistic GDP loss (Euro)**	**N**_**min**_	**N**_**max**_	**Costs/Exam**	**Cost**_**min**_	**Cost**_**max**_
Employed male patients, age < 65 years (n)	895	11,821	26,70	23,904	315,628
Employed female patients, age < 65 years (n)	2099	27,718	26,70	56,048	740,061
Total GDP loss				79,952	1,055,689
**Sonography costs incl. GDP loss**	**–**	**–**	**–**	**154,116**	**2,034,951**

Full cost pricing for liver sonography was based on the median total exam time (12.4 min). Opportunistic costs for diagnostic sonography was based on the median turnaround time (45 min). The total costs were calculated on basis of the estimated cHB prevalence in Germany with antiviral treatment indication (*n* = 13,169-97,393), which was adjusted by the rate of patients (24.7–44.1%) with a PAGE-B score ≤ 9 points. Wage costs and gross domestic productivity (GDP) were adjusted by the unemployment rates of 4.1% for men and 3.3% for women as published by the German federal agency [14]. Unemployed patients were excluded from income and GDP calculations. Income of retired patients (age > 65 years) was assumed to be 15.5% of a working persons income [15], whereas retired patients were excluded from GDP calculation

Given that our full cost calculation may differ from other institutions, we included health insurance reimbursement for an abdominal ultrasound (15.91€/exam according to EBM 33042) to extrapolate HCC screening expenses in Germany [25]. This approach identified a cost reduction (incl. GDP) of 131,704 € up to 1,739,025 € per year and 609,789 € up to 8,051,686 € during a 5-year period, respectively (additional Table 2).

**Fig. 1:**
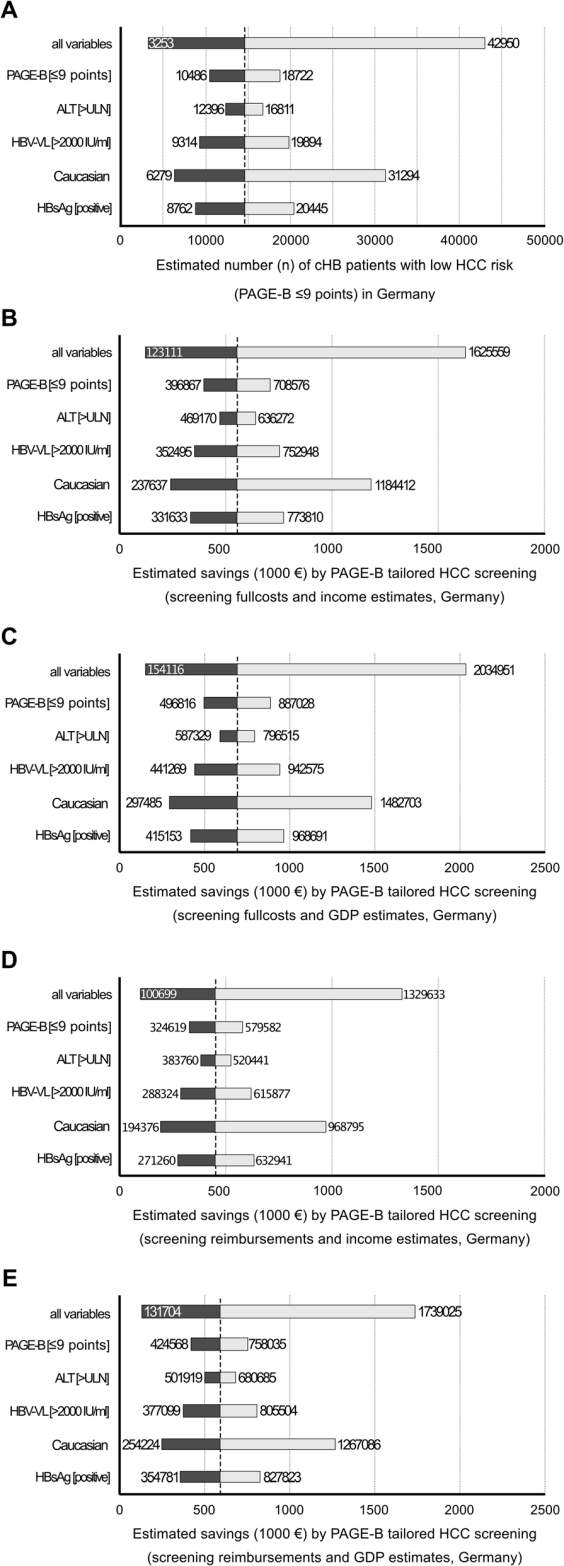
Sensitivity analysis of screening costs for hepatitis B patients (PAGE-B ≤ 9 pts.) in Germany

Factors determining the number of PAGE-B eligible patients in Germany were included into a sensitivity analysis. cHB patient race was also considered, as non-Caucasian patients show a higher overall HCC risk during hepatitis B [26, 27] (Fig. 1a). A comprehensive list of input variables is provided in additional Table 3. Individual sensitivity analyses for HCC screening costs are provided, based on full costs, reimbursements and opportunistic cost due to lost income or lost GDP, respectively (Fig. 1b-e).

### Expenses for omitted HCC diagnosis following PAGE B tailored screening

Postponing HCC screening in the PAGE-B group with low HCC risk could cause costs and a residual health burden by omitted HCC diagnosis. Therefore, indirect costs resulting from missed early HCC diagnosis were extrapolated from the life benefit of HCC screening. Compared to unscreened patients the median survival benefit of HCC screening reaches 5–12 months in cirrhotic patients [28, 29]. The gained life period was subsequently corrected by an average off-work period (94 days per year) caused by the debilitating consequences of malignant diseases [30, 31]. Based on this calculation the median effective time benefit of HCC screening was 473 working hours or 1135 working hours, due to a low (screening interval > 6 months) or a strict screening adherence (screening interval ≤ 6 months), respectively (additional Table 4). Productivity during these time periods eventually resulted in an income benefit of 10,730€ / 25,753€ (low/strict screening adherence) and a GDP benefit of 16,843/40,423€ (low/strict screening adherence) for each HCC diagnosed via screening.

A break-even cost calculation between PAGE-B tailored HCC screening and omitted HCC diagnosis was performed. We applied a screening interval > 6 months in non-cirrhotic cHB patients and included a pooled sensitivity of 86% (95%CI 75–95%) for sonographic detection of any stage HCC as well as a sensitivity of 42% (95%CI 27–58%) for early HCC detection to adjust screenings costs, accordingly [32]. Economic break-even is achieved by PAGE-B tailored screening, if annual HCC incidence does not exceed 0.28% (100% sensitivity), 0.33% (85% sensitivity) and 0.67% (42% sensitivity) in the low HCC risk group. The corresponding GDP break-even calculation identified a maximum annual HCC incidence of 0.12% (100% sensitivity), 0.14% (85% sensitivity) and 0.28% (42% sensitivity) in the low HCC risk group, respectively. Additional institutional cost by omitted HCC diagnosis were not expected, as screening does not prevent HCC occurrence and early diagnosis of localized HCC is associated with higher health care costs [31].

## Discussion

The PAGE-B score was successfully introduced to tailor HCC surveillance in our cHB patient cohort. This included cautious selection of cHB monoinfected patients receiving NA therapy for one year, who are eligible to apply the PAGE-B score. The low risk subgroup defined by the PAGE-B score ≤ 9 points hereby showed no residual HCC risk, as previously identified by different clinical trials [8, 9]. Following this approach at our institution the PAGE-B score reduced annual sonography unit costs by 15.51% for HCC screening of cHB patients. This equals a cost reduction of 1.91% for our annual sonography expenses. In comparison, population-based estimates suggested to postpone HCC screening in 1.35% up to 7.65% of cHB patients in Germany. Given that our referral center for liver diseases reached a high NA therapy uptake of 97% (*n* = 258/266) in cHB patients, the general rate of PAGE-B eligible cHB patients receiving NA treatment could be comparably lower. Particularly, as European and American trials have reported an average NA treatment uptake of only 41% in cHB patients with therapy indication [33].

The presented full cost calculation identified lower expenses for diagnostic sonography compared to published costs of 31.43–51.47 € per exam [34]. These differences result from a shorter exam time (12.4 min) quantified at our institution, compared to the exam time (~ 20 min) assumed by the other authors [35, 36]. A time-related effect particularly holds true, as full costs for diagnostic sonography adjusted to 20 min were 37.18 € per exam, which was in line with a recent German cost calculation [34]. However, the exam time of previous studies was either derived from a limited number of sonographies (*n* = 30) [36] or was assessed by a practitioner questionnaire, which did not include any standardized time acquisition [35]. Therefore, robust data are provided by the presented approach, which employed a reliable time tracking system during the project, avoiding any time lag between the monitored activity and documentation. More so, the study focused on HCC screening in the ambulatory setting, which potentially reduced the average exam time, as shorter exam times for out-patients (18.9 min) compared to hospitalized patients (21.7 min) were observed during sonography [35].

The presented consumable costs were based on a published cost point composition, which covers all aspects of sonography screening [34]. Our assumptions did not include variable instrument expenses between 50,000 € and 125,000 €, depending on the configuration of ultrasonography unit. Instrument expenses may therefore alter costs for a single liver ultrasound to 21.22 € (− 7.02%) and 24.45 € (+ 7.15%). Marginal differences compared to published full cost calculations were also observed for the personnel costs, as medical training at our institution has some effect on personnel related expenses. Despite these observational limitations we provide a precise cost assessment, which is in line with previous cost calculations and could be generalized for the HCC screening in Germany [34].

The total population-based costs for sonographic HCC screening were based on the full costs for liver sonography and productivity loss in patients. Estimates of lost productivity (GDP) (26.7 €/exam) or lost earnings (15.6–17.5 €/exam) hereby dependent on the cross section of the analyzed population [37, 38]. Current German population surveys and census were applied for this project, which do not entirely represent the composition of cHB patients [13, 39]. Hence, data were adjusted for age and gender, as younger female patients assigned to low PAGE-B risk group have a lower income and productivity compared to the average population [13, 16]. Due to limited data, the rate of foreign cHB patients for example and their specific human capital could not be considered. A microcensus showed that among households with a low income (< 500 €/ month) the rate of persons with migration background is 66.2% for example [40]. Given that an immigration status was present in 35.6 up to 60% of HBsAg-positive persons, this has some impact on the estimated human income loss [19, 22].

Analysis of reimbursements for HCC screening seems more reliable for nationwide cost exploration, as reimbursements are not affected by local cost factors. Estimated savings of reimbursement (incl. GDP) by PAGE-B tailored HCC screening were 15.5% lower compared to savings based on full cost calculation (incl. GDP). The driving factor for this difference was lower reimbursement (15.91 €/exam) compared to full costs (22.8 €/exam) for sonography screening, which confirms a funding gap for sonography in Germany [34]. We therefore argue that full cost calculation covers the entire utilization of health care resources for HCC screening, which highlights the value of empiric data provided by our study.

Health burden and consequence costs by omitted HCC diagnosis after PAGE-B risk stratification became a concern, as an Asian cohort revealed a cumulative 5-year incidence of 0.4% in the low HCC risk group [27, 41]. Improved HCC prediction by incorporation of liver function (albumin) into a modified PAGE-B score hereby indicated that unrecognized liver cirrhosis could be responsible for this finding [27, 42]. Additional host factors seem accountable, as the PAGE-B score in mainly Caucasian patients did not omit residual HCC incidence in the low risk group [8–10]. We argue that remaining HCC risk following a PAGE-B stratification is intrinsic to the East-Asian population, who generally harbor a higher HCC burden during chronic B infection [26]. Racial disparities of non-cirrhotic HCC patients with African and Asian descent are particularly relevant [43], as the PAGE-B score relies on surrogate markers of liver cirrhosis (e.g. thrombocyte counts). We therefore conclude that PAGE-B tailored HCC screening should be restricted to cHB patients without liver cirrhosis to minimize consequence costs by omitted HCC diagnoses. In our cHB cohort this was achieved by an initial ultrasound staging or optional liver elastography, which are part of the recommended primary work up of HBV infected patients [3]. Including ethnicity into extrapolation of PAGE-B eligible patients further reduces residual HCC incidence and largely eliminates consequence costs of omitted HCC diagnosis. In fact, eliminating patients with minimal HCC risk would increase HCC incidence in the remaining population. This in turn will raise screening efficacy, as higher pre-screen probability reduces false positive HCC screening results. For example, patient selection by PAGE-B reveals an annual HCC incidence of 0.6 and 3.4% in intermediate and high risk group, respectively [8–10]. Based on a Markov model a quality adjusted year (QALY) gained via HCC screening would cost 110,000$/QALY in the intermediate risk group and only 93,000$/QALY in the high risk group [44]. Reallocation of HCC screening, therefore, contributes to additional cost savings including institutional and health care expenses (additional Fig. 2). More importantly, avoiding false positive HCC screening results, will also reduce risks associated with unneeded diagnostic procedures or interventions for the patients.

We are aware that our data require adjustment by hepatitis B epidemiology, average income, per capita GDP to apply absolute savings of PAGE-B tailored HCC screening to other countries. Foremost reimbursement for HCC screening depends on regional health politics and recourse allocation. Relative cost reduction derived from our study, however, applies to western European countries, which share similar epidemiological features of hepatitis B [23, 45].

Following our analyses, we conclude that PAGE-B risk score adapted HCC screening of Caucasian cHB patients without liver cirrhosis is efficient and safe to reduce costs. Particularly, automated calculation of the PAGE-B score and its readily available components make it a nearly cost neutral tool to reduce sonography expenses. PAGE-B score-based screening allocation to patients deserving HCC surveillance also protects limited personnel resources. Tailored screening could, therefore, focus on high risk populations, still facing suboptimal uptake of HCC surveillance of 28 to 65% [46, 47]. Resource sparing risk assessment therefore combines cost reduction as well improvement of healthcare allocation, particularly in context of a relevant funding gap for HCC surveillance.

Tornado blots showing sensitivity analysis of extrapolated screening costs for hepatitis B patients (PAGE-B ≤ 9 pts.) in Germany. Sensitivity analysis of patient numbers (**A**) eligible for PAGE-B tailored screening was performed. Sensitivity analysis of saved HCC screening full costs, include opportunistic lost income (**B**) or opportunistic lost GDP (**C**). Sensitivity analysis of saved HCC screening reimbursements, include opportunistic lost income (**D**) or opportunistic lost GDP (**E**).

Input variables and their variances are as follows: HBsAg positivity rate in the general population (HBsAg, 0.3–0.7%), rate of Caucasian descent among HBsAg positive patients (85–93%), hepatitis B replication among HBsAg positive patients (HBV-VL > 2000 IU/ml, 14.7–31.4%) and hepatitis among replicative HBsAg positive patients (ALT >ULN, 43.8–59.4%). The dashed center line represents costs resulting from average variables according to source data. HBsAg, Hepatitis B surface antigen; ALT, alanine aminotransferase; HBV-VL, hepatitis B virus load; IU, international units; ULN, upper limit of normal.

## Supplementary Information

**Additional file 1: Suppl. Figure 1:** Work flow at the out-patient clinic. **Suppl. Figure 2:** HCC screening costs in relation to HCC incidence. **Suppl. Table 1**: PAGE-B Score. **Suppl. Table 2**: Screening reimbursements for hepatitis B patients (PAGE-B ≤9 pts.) in Germany. **Suppl. Table 3**: Patients with chronic hepatitis B and antiviral therapy indication (Germany)**. Suppl. Table 4**: Productivity benefit per person and HCC diagnosed via screening**.**

## Data Availability

The dataset analyzed during the current study is available from the corresponding author on reasonable request.

## Abbreviations

cHB: Chronic hepatitis B
HCC: Hepatocellular carcinoma
NA: Nucleos(t)ide analogues
PAGE-B: Platelet-age-gender–HBV risk score
HBsAg: HBs-antigen
ETV: Entecavir
TDF: Tenofovir disoproxil fumarate
TAF: Tenofovir alafenamid fumarate
n: Number
€: Euro
IQR: Interquartile range
ALT: Alanine aminotransferase
AST: Aspartate aminotransferase
g: Gram
kPa: kiloPascal
U: Units
USD: United States Dollar
l: Liter
ml: Milliliter
min: Minute
GDP: Gross domestic productivity
