# Supplementary material for: Cost evaluation of PAGE-B risk score guided HCC surveillance in patients with treated chronic hepatitis B
Source: BMC Health Serv Res. 2021 Aug 21;21:846. doi: 10.1186/s12913-021-06794-6 (PMC8379870; doi:10.1186/s12913-021-06794-6)
Supplement: Supplementary file 1 — Additional file 1: Suppl. Figure 1: Work flow at the out-patient clinic. Suppl. Figure 2: HCC screening costs in relation to HCC incidence. Suppl. Table 1: PAGE-B Score. Suppl. Table 2: Screening reimbursements for hepatitis B patients (PAGE-B ≤9 pts.) in Germany. Suppl. Table 3: Patients with chronic hepatitis B and antiviral therapy indication (Germany). Suppl. Table 4: Productivity benefit per person and HCC diagnosed via screening. [file 12913_2021_6794_MOESM1_ESM.docx]

**Suppl. Figure 1:** Work flow at the out-patient clinic

**
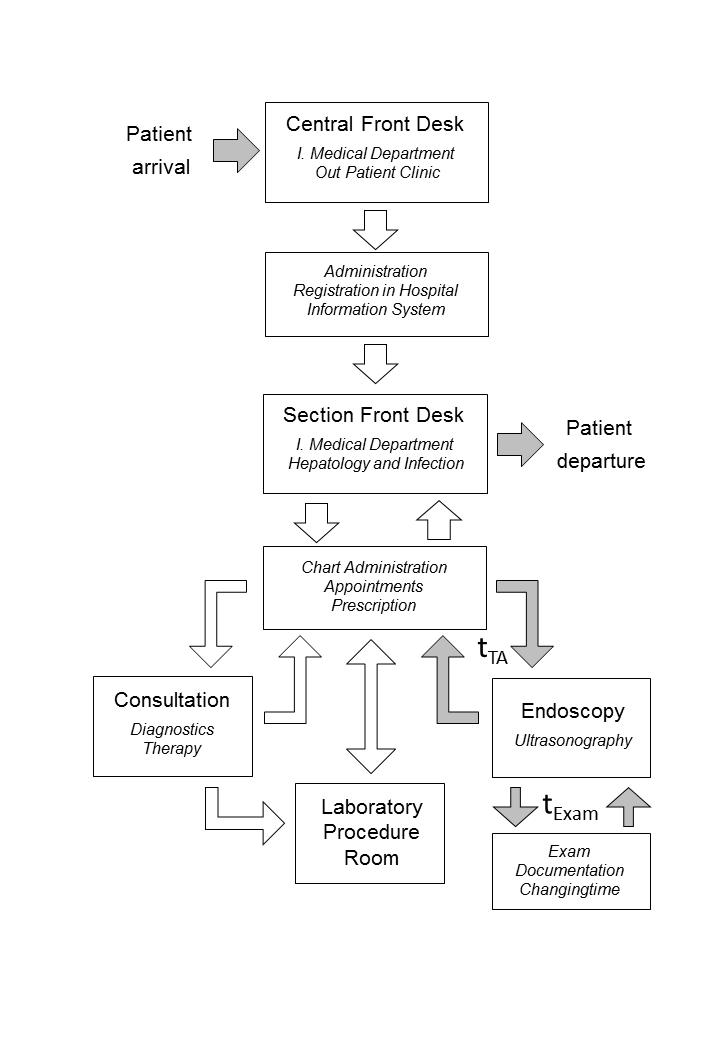
**

**Suppl. Figure 1:** Work flow at the out-patient clinic. The infrastructure and rooms are given in normal letters, whereas services and procedures are listed in *italic* letters. The patients´ turnaround time spend at the endoscopy ward (t_TA_) was documented at the front desk of the out-patient clinic. The time during ultrasonography (t_Exam_) was documented by the examiner via a Timeulite^®^ device.

**Suppl. Figure 2:** HCC screening costs in relation to HCC incidence

#

**Suppl. Figure 2**: Relation of the HCC incidence in the screened population and costs (USD, US. dollars) for a quality adjusted life year (QALY) gained by HCC screening. The graph was derived from the Markov cost model published by Parikh et al. [1]

**Suppl. Table 1:** PAGE-B Score

| **Age** (years) | **Gender** (male/female) | **Platelet count** (n/mm³) |
| --- | --- | --- |
| 16 – 29: 0 pts. | Female: 0 pts. | >200.000: 0 pts. |
| 30 – 39: 2 pts. | Male: 6 pts. | 100.000 – 199,999: 6 pts. |
| 40 – 49: 4 pts. |  | <100,000: 9 pts. |
| 50 – 59: 6 pts. |  |  |
| 60 – 69: 8 pts. |  |  |
| ≥70: 10 pts. |  |  |
| PAGE-B Score according to G. Papatheodoridis *et al.* [2] to assess HCC risk in Caucasian cHB patients under treatment with NAs (entecavir or tenofovir). Patients are categorized into a low (≤9 pts.), medium (10–17 pts.) and high (≥18 pts.) HCC risk group. The score ranges from 0- 25 points (pts.) | | |

Suppl. Table 2: Screening reimbursements for hepatitis B patients (PAGE-B ≤9 pts.) in Germany

| **PAGE-B eligible cHB cohort (PAGE-B score ≤9** **points)** | | | | | |
| --- | --- | --- | --- | --- | --- |
|  | **N_min_** | **N_max_** | **Costs/Exam** | **Cost_min_** | **Cost_max_** |
| **Sonography reimbursement (Euro)** | 3253 | 42950 | 15.9 | 51752 | 683336 |
| **Opportunistic wage costs (Euro)** | N**_min_** | N**_max_** | Costs/Exam | Cost**_min_** | Cost**_max_** |
| **Male patients (n)** | 934 | 12327 | --- | --- | --- |
| Employed male patients (n) | 895 | 11821 | --- | --- | --- |
| Employed male patients, age <65 years (n) | 895 | 11821 | 17.50 | 15667 | 206872 |
| **Female patients (n)** | 2319 | 30623 | --- | --- | --- |
| Employed female patients (n) | 2243 | 29613 | --- | --- | --- |
| Employed female patients, age <65 years (n) | 2099 | 27718 | 15.70 | 32957 | 435167 |
| Employed female patients, age >65 years (n) | 134 | 1774 | 2.40 | 322 | 4257 |
| Total wage costs | --- | --- | --- | 48947 | 646297 |
| **Sonography reimbursement incl. opportunistic wage loss** | **---** | **---** | **---** | **100699** | **1329633** |
| **Opportunistic GDP loss (Euro)** | **N_min_** | **N_max_** | **Costs/Exam** | **Cost_min_** | **Cost_max_** |
| Employed male patients, age <65 years (n) | 895 | 11821 | 26.70 | 23904 | 315628 |
| Employed female patients, age <65 years (n) | 2099 | 27718 | 26.70 | 56048 | 740061 |
| Total GDP loss |  |  |  | 79952 | 1055689 |
| **Sonography reimbursement incl. GDP loss** | **---** | **---** | **---** | **131704** | **1739025** |
| Health insurance reimbursement for an abdominal ultrasound (15.91€/exam) was applied [3]. Opportunistic costs for diagnostic sonography was based on the median turnaround time (45 minutes). The total costs were calculated on basis of the estimated cHB prevalence in Germany with antiviral treatment indication (n=13,169-97,393), which was adjusted by the rate of patients (24.7%-44.1%) with a PAGE-B score ≤9 points. Wage costs and gross domestic productivity (GDP) were adjusted by the unemployment rates of 4.1% for men and 3.3% for women as published by the federal agency, Germany [4]. Unemployed patients were excluded from income and GDP calculations. Income of retired patients (age > 65 years) was assumed to be 15.5% of a working persons income [5], whereas retired patients were excluded from GDP calculation. | | | | | |

Suppl. Table 3: Patients with chronic hepatitis B and antiviral therapy indication (Germany)

| **Dependent population strata** | **Factor** | **Patient (N)** | **Reference** |
| --- | --- | --- | --- |
| Total population (Germany) | baseline | 80,209,997 | [6] |
| HBsAg positive | 0.3%-0.7% | 240,629-561,469 | [7-11] |
| Caucasian | 85%-93% | 204,535-522,167 | [9] |
| HBV viral load (>2000 IU/ml) | 14.7%-31.4% | 30,067-163,960 | [8, 12] |
| Elevated ALT (>ULN) | 43.8%-59.4% | 13,169-97,393 | [8, 9] |
| PAGE-B score ≤9 points | 24.7%-44.1% | 3,253-42,950 | [2, 13] |
| *The total German population was derived from the population census (2011) and modeled for the year 2018. The total population included persons >15 years of age. IU, international units; ULN, upper limit of normal.* | | | |

Suppl. Table 4: Productivity benefit per person and HCC diagnosed via screening

|  | **No HCC screening** | **HCC Screening**  **(interval >6 months)** | **HCC Screening**  **(interval ≤6 months)** | **Reference** |
| --- | --- | --- | --- | --- |
| Median OS (median) | 5 | 10 | 17 | [14, 15] |
| Benefit OS (median) | 0 | 5 | 12 | --- |
| Benefit working hours | --- | 746 | 1790 | --- |
| Loss working hours by HCC | --- | 273 | 654 | [16, 17] |
| Effective Working hours benefit | --- | 473 | 1135 | --- |
| Income benefit per screened HCC (€) | --- | 10730 | 25753 | --- |
| Productivity (GDP) benefit per screened HCC (€) | --- | 16843 | 40423 | --- |
| Working time benefit by HCC diagnosed by screening and resulting gained productivity were calculated. | | | | |

# References

1. Parikh ND, Singal AG, Hutton DW, Tapper EB: **Cost-Effectiveness of Hepatocellular Carcinoma Surveillance: An Assessment of Benefits and Harms**. *The American journal of gastroenterology* 2020, **115**(10):1642-1649.

2. Papatheodoridis G, Dalekos G, Sypsa V, Yurdaydin C, Buti M, Goulis J, Calleja JL, Chi H, Manolakopoulos S, Mangia G *et al*: **PAGE-B predicts the risk of developing hepatocellular carcinoma in Caucasians with chronic hepatitis B on 5-year antiviral therapy**. *Journal of hepatology* 2016, **64**(4):800-806.

3. **Einheitlicher Bewertungsmaßstab (EBM)** [<https://www.kbv.de/html/ebm.php>]

4. **Genesis-Online database: Unemployment** [<https://www.destatis.de/DE/Themen/Arbeit/Arbeitsmarkt/Erwerbslosigkeit/_inhalt.html>]

5. Johnson RW, Schaner SG: **Value of unpaid activities by older americans tops $160 billion per year**. In: *The retirement project.* vol. 2. Washington: The urban institute; 2005.

6. Bundesamt S: **Bevölkerung (Zensus), nach Bundesländer, Stichtag, Nationalität, Altersgruppen**. 2018.

7. **HBV nach Jahr und Falldefinition 2001 bis 2018** [<https://survstat.rki.de/Content/Query/Create.aspx>]

8. Wolffram I, Petroff D, Batz O, Jedrysiak K, Kramer J, Tenckhoff H, Berg T, Wiegand J, German Check-Up 35+ Study G: **Prevalence of elevated ALT values, HBsAg, and anti-HCV in the primary care setting and evaluation of guideline defined hepatitis risk scenarios**. *Journal of hepatology* 2015, **62**(6):1256-1264.

9. Fischer C, Mauss S, Zehnter E, Bokemeyer B, Heyne R, Huppe D: **[Epidemiology and clinical characteristics of patients with chronic hepatitis B (CHB) in Germany - results of a nationwide cross-sectional study]**. *Z Gastroenterol* 2012, **50**(1):22-29.

10. Huetter ML, Fuchs M, Hanle MM, Mason RA, Akinli AS, Imhof A, Kratzer W, Lorenz R, group Es: **Prevalence of risk factors for liver disease in a random population sample in southern Germany**. *Z Gastroenterol* 2014, **52**(6):558-563.

11. Poethko-Muller C, Zimmermann R, Hamouda O, Faber M, Stark K, Ross RS, Thamm M: **[Epidemiology of hepatitis A, B, and C among adults in Germany: results of the German Health Interview and Examination Survey for Adults (DEGS1)]**. *Bundesgesundheitsblatt Gesundheitsforschung Gesundheitsschutz* 2013, **56**(5-6):707-715.

12. Tan M, Bhadoria AS, Cui F, Tan A, Van Holten J, Easterbrook P, Ford N, Han Q, Lu Y, Bulterys M *et al*: **Estimating the proportion of people with chronic hepatitis B virus infection eligible for hepatitis B antiviral treatment worldwide: a systematic review and meta-analysis**. *Lancet Gastroenterol Hepatol* 2021, **6**(2):106-119.

13. Riveiro-Barciela M, Tabernero D, Calleja JL, Lens S, Manzano ML, Rodriguez FG, Crespo J, Piqueras B, Pascasio JM, Comas C *et al*: **Effectiveness and Safety of Entecavir or Tenofovir in a Spanish Cohort of Chronic Hepatitis B Patients: Validation of the Page-B Score to Predict Hepatocellular Carcinoma**. *Dig Dis Sci* 2017, **62**(3):784-793.

14. Choi DT, Kum HC, Park S, Ohsfeldt RL, Shen Y, Parikh ND, Singal AG: **Hepatocellular Carcinoma Screening Is Associated With Increased Survival of Patients With Cirrhosis**. *Clin Gastroenterol Hepatol* 2019, **17**(5):976-987 e974.

15. Zhang BH, Yang BH, Tang ZY: **Randomized controlled trial of screening for hepatocellular carcinoma**. *Journal of cancer research and clinical oncology* 2004, **130**(7):417-422.

16. Lang K, Danchenko N, Gondek K, Shah S, Thompson D: **The burden of illness associated with hepatocellular carcinoma in the United States**. *Journal of hepatology* 2009, **50**(1):89-99.

17. Yabroff KR, Lawrence WF, Clauser S, Davis WW, Brown ML: **Burden of illness in cancer survivors: findings from a population-based national sample**. *J Natl Cancer Inst* 2004, **96**(17):1322-1330.
